# Tea Tree Oil Improves Energy Metabolism, Non-Specific Immunity, and Microbiota Diversity via the Intestine–Hepatopancreas Axis in *Macrobrachium rosenbergii* under Low Fish Meal Diet Administration

**DOI:** 10.3390/antiox12101879

**Published:** 2023-10-19

**Authors:** Mingyang Liu, Xiaodi Xu, Cunxin Sun, Xiaochuan Zheng, Qunlan Zhou, Changyou Song, Pao Xu, Qiang Gao, Bo Liu

**Affiliations:** 1 Wuxi Fisheries College, Nanjing Agricultural University, Wuxi 214081, China; liumy013@163.com (M.L.); 2021213005@stu.njau.edu.cn (X.X.); suncx@ffrc.cn (C.S.); zhouql@ffrc.cn (Q.Z.); 2 Key Laboratory of Freshwater Fisheries and Germplasm Resources Utilization, Ministry of Agriculture and Rural Affairs, Freshwater Fisheries Research Center, Chinese Academy of Fishery Sciences, Wuxi 214081, China; zhengxiaochuan@ffrc.cn (X.Z.); songchangyou@ffrc.cn (C.S.); 3 Key Laboratory of Healthy Freshwater Aquaculture, Ministry of Agriculture and Rural Affairs, Key Laboratory of Fish Health and Nutrition of Zhejiang Province, Zhejiang Institute of Freshwater Fisheries, Huzhou 313001, China

**Keywords:** *M. rosenbergii*, alternative fish meal diet, plant essential oil, intestinal microbes, energy metabolism, crustacean immunity

## Abstract

Tea tree oil (TTO) is an essential plant oil with diverse antibacterial and antioxidant properties; however, whether the role played by TTO in low fish meal (LF) diets induced the observed effects in the farmed crustaceans remains unclear. Therefore, this study used *Macrobrachium rosenbergii* as the model crustacean, and an 8-week feeding experiment with NF (normal fish meal), LF (soybean meal replacing 40% fish meal), and LFT (LF with 200 mg/kg TTO) diets was conducted to evaluate the positive effects of TTO under the LF diet. Compared to the NF diet, the LF diet reduced hemolymph antioxidant capacity and non-specific immunity, and induced hepatopancreas apoptosis and damage. However, in comparison with LF, LTF significantly ameliorated morphological impairment in the hepatopancreas, improved hepatopancreas energy metabolism by upregulating the *Bcl-2*/*Bax* and *Akt*/*mTOR* pathways, and enhanced antioxidant and non-specific immune capacity by activating the *NF-κB*/NO pathway. In addition, LFT repaired intestinal barrier injury and the imbalance of intestinal microbiota induced by the LF diet. Moreover, the Pearson correlation revealed the variations of the above indicators, which were related to the abundance changes of *Klebsiella*, *Clostridium sensu stricto 12*, *Thermobifida*, *Bifidobacterium*, and *Alistipes*, indicating that these microbes might serve as prospective targets for the intestine–hepatopancreas axis to affect hepatopancreas apoptosis, metabolism, and non-specific immunity. In summary, 200 mg/kg TTO supplementation mediated gut microbiota and positively improved energy metabolism and non-specific immunity, thereby alleviating hepatopancreas dysplasia and damage induced by the LF diet in *M. rosenbergii*.

## 1. Introduction

With the flourishing development of the aquaculture industry, the demand for fish meal in the formulation of aquatic feeds is gradually increasing, causing the price of fish meal to rise rapidly [1]. Therefore, it is necessary to explore inexpensive but effective plant protein foodstuffs that could partially replace fish meal in aquatic feeds. Soybean meal (SBM) is the most common alternative protein source in aquatic feed because of its high protein content, favorable price, balanced amino acid profile, and high level of digestibility. However, high levels of SBM have been shown to contain high doses of anti-nutritional factors (ANFs), which result in adverse effects including reduced growth performance and oxidative stress [2]. Under clean-water culture systems, as observed in several studies, SBM could generally replace 37–56% of dietary fish meal in the feed to achieve a non-influential growth performance for shrimp, and other plant protein sources were equal to or less efficient than soybean meal [3]. However, another research study showed that when SBM replaced 30% fish meal, it induced a downregulation of the growth performance and non-specific immunity in *Litopenaeus vannamei* [4]. Based on these studies, this experiment used SBM instead of 40% fish meal to create a low fish meal negative model.

The hepatopancreas is the major energy storage organ and a critical hematopoietic and energy metabolic organ in crustaceans [5]. Replacing fish meal with vegetable protein in low fish meal diets resulted in hepatopancreatic damage (increased hepatocyte necrosis and apoptosis), impaired development (downregulated expression of *IGF-1* and *IGF-2* signaling factors), and a decreased hepatosomatic ratio [6,7]. This may be due to impaired energy metabolism in the hepatopancreas [8,9]. Low fish meal diets substituted with plant proteins also affected intestinal barrier function and intestinal health [10]. Furthermore, soybean meal’s replacement of fish meal was found to cause host soybean meal-type enteritis, which manifests as intestine structural damage and the downregulation of immunity associated with the *Toll*-*NF-κB* signaling pathway [11], which is reported to be caused by intestinal flora and their metabolite alternation [12]. Gut microorganisms are essential in the gut–liver axis, which is involved in the homeostasis of digestion and absorption, the maintenance of the intestinal barrier, the regulation of immunity, and regulation of liver function [13]. Research indicates that intestinal flora imbalance can induce hepatopancreas damage and metabolic disorders [14]. The imbalance of intestinal flora leads to intestinal barrier damage by increasing intestinal permeability, which leads to pathogenic bacteria infection and associated harmful metabolite (endotoxin) accumulation, thereby increasing the occurrence and progression of liver damage through intestine–liver axis circulation [15]. Meanwhile, some metabolites of beneficial gut microbes, such as short-chain fatty acids (SCFAs) and bile acids (BAs), could enter the liver via enterohepatic circulation and positively regulate hepatic metabolism and energy homeostasis [16]. Furthermore, in crustaceans the gut microbiota also shows significant positive or negative correlations with many biomarkers of hepatopancreatic injury, suggesting that the gut microbiota is involved in hepatopancreatic injury [17]. Therefore, gut microbes should be considered when assessing the hepatopancreatic health of crustaceans.

In general, plant-derived feed additives have been used to alleviate growth inhibition and metabolic diseases in aquaculture because of their nonpolluting and highly efficient properties [18]. Tea tree oil (TTO) is an essential oil obtained from *Melaleuca alternifolia* via steam distillation. Due to its active antioxidant component, 4-terpineol, TTO could scavenge excess reactive oxygen species (ROS) and enhance the antioxidant capacity of the host [19]. Our previous study found that tea tree oil alleviated oxidative stress by activating the *NF-κB*/NO pathway [20]. Meanwhile, plant essential oils have been found to improve the intestinal flora structure, inhibit the growth of harmful bacteria, and enhance the colonization ability of beneficial bacteria in the intestine [21,22]. In invertebrates, plant essential oils have been found to inhibit the abundance of *Proteobacteria* and increase the abundance of the beneficial bacteria *Lactobacillaceae* [23]; however, the mechanism of action is unclear.

*Macrobrachium rosenbergii* is cultivated worldwide and brings high economic value, and its production reached 177,836 tons with a market value of CNY 10 billion in China in 2022 [24]. In addition, *M. rosenbergii* is an important research model for crustacean nutrition, metabolism, and immunology, and has significant scientific research value. Also, there have been limited studies on health and immunomodulation from the perspective of the hepatopancreas–intestine axis in crustaceans, which is a valid area of research and one of great significance. Therefore, *M. rosenbergii* could be used as an invertebrate model to develop low fish meal diets and to study the mechanism of the hepatopancreas–intestine axis. In view of this, our research aims to investigate and evaluate the protective effects of TTO (200 mg/kg, Figures S1 and S2) [25] on the metabolism, antioxidant, immunity, and intestinal microflora diversity of *M. rosenbergii* against the side effects induced by a low fish meal diet (soybean meal replacement).

## 2. Materials and Methods

### 2.1. Experimental Animals and Ethical Statement

Batches of *M. rosenbergii* prawns (0.28 ± 0.01 g body weight) were obtained from Zhejiang South Taihu Lake Freshwater Aquatic Seed Industry Co., Ltd., Huzhou, China. For temporary breeding, prawns were maintained for 7 days in three aerated cylindrical polypropylene tanks (100 cm height × 180 cm diameter) and fed with commercial feed (Fuyuda Food Products Co., Ltd., Yangzhou, China) before feeding experiments.

All experiments were conducted in accordance with the guidelines for the scientific breeding and use of animals from the Institutional Animal Care and Use Committee (IACUC) of the CAFS. At the same time, the animal care and use regulations of Nanjing Agricultural University were followed.

### 2.2. Experimental Design and Rearing Conditions

The experimental diets were formulated and presented in Table S1. Briefly, three experimental diets were used: the normal fish meal diet (NF), the low fish-meal diet (LF), and the low fish-meal diet supplemented with 200 mg/kg TTO (LFT). The TTO emulsion (10% available content) was purchased from Nanjing Shanghao Technology Co., Ltd. (Nanjing, China).

A total of 360 shrimps were weighed using an electronic scale with a precision of 0.01 (body weight: 0.28 ± 0.01 g) and then stocked into 9 polypropylene tanks. As in the temporary culture, each tank was interconnected with the recirculating water filtration system (supplied by Zhongke Seawater Treatment Co., Ltd., Qingdao, China), and the chlorine-free water treated by the filtration system filled half of each tank. A total of 40 prawns per tank and 9 tanks were randomly assigned to three experimental diet groups, with 3 replicates. As the experiment was conducted indoors, 10 h of light exposure per day had to be provided by means of large lamps. The temperature, pH and dissolved oxygen were 29–33 °C, 7.5–8.4, and >5 mg/L. Prawns were fed three times a day for 8 weeks at 8:00, 13:00, and 18:00 to apparent satiation. After 1 h of feeding, the remaining feed was collected using an 80-mesh sieve and weighed on an electronic scale to determine the feed conversion ratio. The experiment lasted 8 weeks and sampling and measurements were performed at the end of the experiment.

### 2.3. Growth Evaluation

After an 8-week feeding experiment, the survival rates, final weights of prawns, and the hepatopancreases weight were recorded in each group (Table S2). The weight gain rate, specific growth rate, feed conversion ratio, and hepatosomatic index were calculated using the following methods:

- Weight gain rate (WGR, %) = (Final weight (g) − Initial weight (g))/initial weight × 100;
- Specific growth rate (SGR, %/day) = (Ln final weight − Ln initial weight) × 100/days;
- Feed conversion ratio (FCR) = Dry feed intake (g)/weight gain (g);
- Hepatosomatic index (HSI, %) = (Hepatopancreas weight/final weight) × 100.

### 2.4. Samples Collection

After an 8-week feeding trial, all sampled prawns were fasted for 24 h to empty the digestive tract, and 18 prawns were randomly selected in each group. First, the Elsevier solution (13.2 g/L trisodium citrate, 4.8 g/L citric acid, and 14.7 g/L glucose) was used as an anticoagulant to collect the prawns’ hemolymphs. According to the method of Rodriguez et al., the air was expelled from the 1 mL syringe, 200 μL of anticoagulant drawn, and then the hemolymph extracted from the thorax of the prawns, and the proportion of hemolymph to the anticoagulant was 1:1 [26]. The mixtures were then centrifuged at 4000 rpm for 10 min at 4 °C to separate the hemolymph from blood cells, and the supernatants of 3 prawns in each replicate were placed in a 1.5 mL centrifuge tube. Next, prawns were dissected to obtain hepatopancreas and chyme samples. The hepatopancreases from 3 prawns were randomly mixed in each replicate and put into a 2 mL cryogenic vial, and the chyme was stored similarly. These cryogenic vials were snap-frozen in liquid nitrogen and finally stored at −80 °C for further analysis. In addition, three hepatopancreases from each diet were randomly collected and fixed in 4% paraformaldehyde and 2.5% glutaraldehyde solution (Macklin Biochemical Co., Ltd., Shanghai, China) for H&E and TEM analysis, respectively. The remaining shrimp should be immediately stored in a freezer at −80 °C to supplement the insufficient test samples.

### 2.5. Biochemical Parameters Analysis

The levels of hemolymph glucose (GLU), triglyceride (TG), total cholesterol (TC), low-density lipoprotein cholesterol (LDL-C), high-density lipoprotein cholesterol (HDL-C), total protein (TP), albumin (ALB), globulin (GLB), aspartate aminotransferase (AST), alanine aminotransferase (ALT), and alkaline phosphatase (AKP) were measured with an automatic hemolymph biochemical analyzer (Mindray BS-400, Shenzhen, China) using 300 µL of hemolymph according to the manufacturer’s instructions with the appropriate commercial kit (Mindray Medical International Co., Ltd., Shenzhen, China). The pretreated hepatopancreas AST, ALT, TP, TG, and TC were measured using Nanjing Jiancheng Institute commercial bioengineering kits. The pre-treatment consisted of homogenising hepatopancreas samples from cryogenic vials in ice-cold saline, centrifuging at 4000 rpm for 10 min at 4 °C, and retaining the supernatant for analysis. In addition, lipopolysaccharide (LPS) was determined using ELISA kits that were specific for prawns (Shanghai mlbio Biotechnology Co., Ltd., Shanghai, China).

### 2.6. Enzyme Activity Analysis

Following the methods used in our previous research [27], total superoxide dismutase (T-SOD), catalase (CAT), glutathione (GSH), and lysozyme (LZM) Nanjing Jiancheng Institute commercial bioengineering kits were used to measure the activity in the hemolymph. The enzyme activity analysis was performed using a Spectra Max Plus spectrophotometer (Molecular Devices, Menlo Park, CA, USA), monitored at 450 nm, 550 nm, 420 nm, and 530 nm wavelengths, respectively. The concentration of malondialdehyde (MDA) in the hemolymph was determined using the thiobarbituric acid (TBA) method and measured at 532 nm [25]. A pre-processing step is required prior to the detection of hepatopancreas. As is general, the liver and pancreas were suspended in a 50 mM potassium phosphate buffer containing 0.5 mM EDTA at a pH of approximately 7.0. The suspension was homogenized using a handheld homogenizer for 5 min in a 2.0 mL centrifuge tube. After homogenization, all samples were centrifuged at 4 °C for 15 min at a speed of 2000× *g*. The supernatant was then extracted for subsequent enzyme activity analysis. The hepatopancreatic T-SOD, CAT, GSH, and MDA were then determined in the same way as the hemolymph parameters. The hepatopancreatic amylase (AMS) and trypsin (TPS) were analyzed using commercial bioengineering kits from Beijing Solarbio Co., Ltd. (Beijing, China) at 540 nm and 253 nm, respectively.

### 2.7. H&E, TUNEL, and Oil Red O Staining, and TEM Analysis

Hepatopancreas samples fixed in 4% paraformaldehyde buffer were removed. The samples were embedded in optimal cutting temperature (OCT) medium and stored at −80 °C. According to our published work [27], hematoxylin-eosin (H&E) and Oil Red O (ORO) staining of the hepatopancreas was performed and microimaging was performed using a Leica DM1000 optical microscope (Wetzlar, Germany) light microscope. Hepatocyte apoptosis was determined with Xu et al.’s methods and using the terminal deoxynucleotidyl transferase-mediated dUTP-biotin nick end labeling (TUNEL) assay [28]. The TUNEL sections were washed and photographed under a fluorescence microscope. ImageJ software 1.52a (National Institutes of Health, Bethesda, MD, USA) was used to analyze the lipid content of the ORO-stained section and positive cells of the TUNEL staining. Intestinal tissue was extracted from the previously prepared 2.5% glutaraldehyde fixative and processed according to Xie et al.’s method for tissue sectioning [13]. The cellular structure was then observed using transmission electron microscopy (Hitachi HT7700 TEM, Tokyo, Japan).

### 2.8. Bacterial 16S rDNA Gene Amplification, cDNA Library Construction, and Sequencing

To profile the diversity and structure of the microbial communities, the 16S rDNA V3-V4 region of the ribosomal RNA gene was amplified with PCR (95 °C for 2 min, followed by 27 cycles at 98 °C for 10 s, 62 °C for 30 s, and 68 °C for 30 s and a final extension at 68 °C for 10 min) using primers 338F: ACTCCTACGGGAGGCAGCAG; 806R: GGACTACHVGGGTWTCTAAT [29], where the barcode is an eight-base sequence unique to each sample. PCR reactions were performed in triplicate 50 μL mixtures, with the specific content determined according to the method of Xue et al. [30]. All PCR products were extracted from 2% agarose gels and purified using the Merck DNA Gel Extraction Kit (Merck Sigma-Aldrich, Darmstadt, Germany) according to the manufacturer’s instructions and quantified using the Bio-Rad CFX96 Real-Time PCR System (Bio-Rad Laboratories, Inc., Hercules, CA, USA). Purified PCR amplicons were pooled in equimolar and paired-end sequenced (2 × 250) on an Illumina platform according to standard protocols and the manufacturer’s instructions.

Clean reads were obtained using the following steps. The barcode and linker sequences were removed and combined with the paired-end reads to form a longer fragment. Reads with average quality scores of <20 and >100 bp were then removed. Mismatching primer sequences or ambiguous bases (Ns) greater than 5% were removed from the downstream analyses. Reads that could not be assembled were discarded. Spliced paired-ended sequences were generated using FLASH (Fast Length Adjustment of SHort reads) [31]. VSEARCH 2.21.1 was used to remove chimeric sequences [32]. Sequences were then classified into operational taxonomic units (OTUs) at the 97% sequence similarity level using UPARSE (version 7.1) [33]. Taxonomic classifications were annotated in the RDP database.

Microbial variation was compared using multiple analytical methods. The Stamp was used to analyze the difference in microbial abundance at the genus level among three diets. The Stamp figure was performed on the Tutools platform http://www.cloudtutu.com (accessed on 18 March 2023).

LEfSe (Linear discriminant analysis Effect Size) was used to analyze the intestinal tract microbial communities under different diets. Briefly, the non-parametric factorial Kruskal–Wallis (KW) sum rank test was used to detect the characteristics of significant differences in abundance and to find the groups with significant differences in abundance. LEfSe linear discriminant analysis (LDA) was used to estimate the impact of each intestinal microbial (species) abundance on the difference effect (screening criteria were *p* < 0.05, LDA score > 3). LEfSe was performed using the OmicStudio tools (www.omicstudio.cn/tool (accessed on 18 March 2023)).

### 2.9. Quantitative Real-Time RT-PCR (qPCR) Validation

According to the hepatopancreas transcriptome data in our lab, the primers for RT-PCR were designed using Primer 5.0 (shown in Table S3).

The total RNA of 6 hepatopancreas in each group was extracted with RNAiso Plus (TaKaRa, Shiga, Japan). Then, RNA concentration was determined with Nanodrop 2000 (Thermo Fisher Scientific, Waltham, MA, USA). The RNA concentration of each sample was diluted to 400 ng/mL, and the first-strand cDNA was generated from 400 ng DNase-treated RNA using a HiScript III 1st Strand cDNA Synthesis Kit (Vazyme Biotech Co., Ltd., Nanjing, China). The Two-Step SYBR^®^ Prime Script^®^ Plus RT-PCR Kit (TaKaRa, Kusatsu City, Japan) was used to perform RT-PCR with a Bio-Rad CFX96 (Bio-Rad Laboratories, Inc., Hercules, CA, USA) real-time PCR system [27], and the 2^−∆∆^CT method was used to calculate the relative gene expression with the internal reference gene β-actin.

### 2.10. Western Blot Analysis

Western blotting was performed to measure protein expression, as described in our previous report [34]. Briefly, tissue protein extraction and concentration determination were performed using RIPA lysis buffer (Epizyme, Shanghai, China), phenylmethanesulfonyl fluoride (Epizyme), SDS-PAGE protein sample loading buffer (Epizyme), and a BCA protein assay kit (Beyotime, Shanghai, China). SDS-PAGE separated protein samples and the protein was transferred to a PVDF membrane. The PVDF membrane was then blocked with BSA (5%) for 2 h at room temperature (RT) and washed three times for 10 min each time. The membranes were then blocked with primary antibodies for 12 h at 4 °C. After equilibration at room temperature for 1 h, the membranes were washed three times for 10 min each and incubated with a secondary antibody solution (goat anti-rabbit horseradish peroxidase conjugate, Santa Cruz Biotechnology, Santa Cruz, CA, USA) for 1 h at RT. An electrochemiluminescence (ECL) kit (Beyotime, P0018FM, China) was used to visualize the immune complexes, and ImageJ software 1.52a was used for quantification. Specific primary antibodies used in this experiment, including Bcl-2 (1:2000), Bax (1:4000), p-AKT (1:5000), AKT (1:2000), NF-κB (1:1000), and β-actin antibody (1:10,000), were purchased from Abcam Biotechnology Ltd. (Cambridge, UK).

### 2.11. Statistical Analysis

All experimental results were expressed as mean ± S.E.M. (standard error of the mean). Student’s *t*-test was used to compare three groups, and comparison between three groups was carried out using one-way ANOVA. The SPSS 15.0 (SPSS Inc., Michigan Avenue, Chicago, IL, USA) was used for the statistical analysis. The significance was set at *p* < 0.05, and extreme significance was set at *p* < 0.01. In addition, normalization was performed on all RT-PCR and WB results. A Pearson correlation analysis was used to analyze the correlation between the two variables that conformed to the normality, using the following marks: one asterisk (*) indicated a significant difference (*p* < 0.05), and two asterisks (**) indicated an extremely significant difference (*p* < 0.01).

## 3. Results

### 3.1. Growth Performance

As shown in Table 1, WGR, SGR, and HSI showed a decreasing followed by an increasing trend among the three groups, and FCR showed a contrary tendency. SR, WGR, SGR, and HSI in the LF group were significantly lower than those in the NF group (*p* < 0.05); however, WGR, SGR, and HSI in the LF group were significantly higher than those in the NF group (*p* < 0.05). In addition, FCR was significantly lower in the LF group than in the NF and LFT groups (*p* < 0.05). The LFT group had no significant difference from the NF group (*p* > 0.05).

### 3.2. TTO Ameliorates Hepatopancreatic Injury from the LF Diet

**Figure 1:**
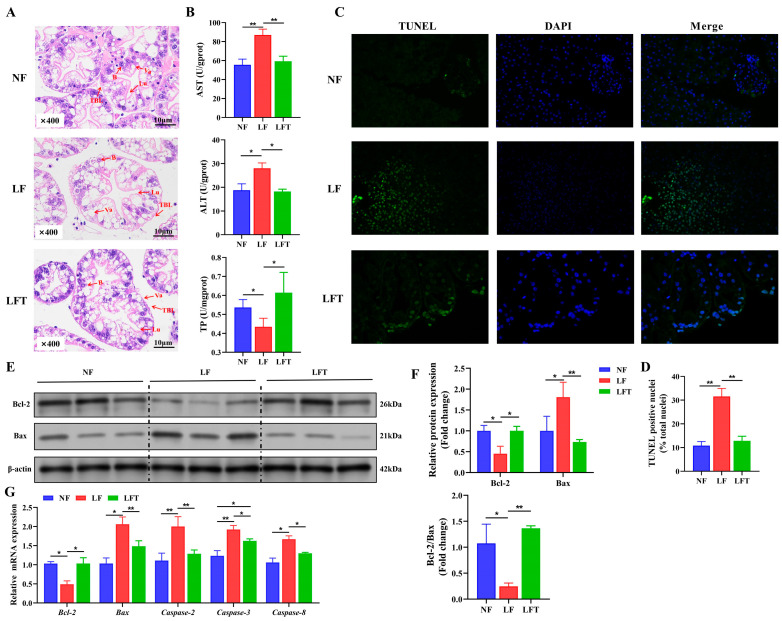
Effect of the three diets on the hepatopancreatic morphology, health, and apoptosis status of *M. rosenbergii*. (**A**) The representative micrographs of H&E stainings (400× magnification); B, secretory cells; Lu, lumen; Va, vacuole; TBL, the thickness of basal laminae. (**B**) The indicators of hepatopancreatic function; AST, aspartate aminotransferase; ALT, alanine transaminase; TP, total protein. (**C**,**D**) The representative micrographs of TUNEL stainings (400× magnification) and percentage of apoptotic nuclei. (**E**,**F**) Western blot assays of Bcl-2 and Bax protein expression and ratio of Bcl-2/Bax. (**G**) mRNA expression of apoptosis-related genes. The asterisk indicated significant differences between the two groups. * *p* < 0.05, ** *p* < 0.01.

The LF group significantly affected the hepatic morphology, as shown in Figure 1A. Visually, the LF group showed a significant decrease in secretory cells and an increase in vacuoles compared to the NF group. There was no significant difference between the NF and LFT groups except for a slight increase in vacuoles. The structures of the lumen in the three groups were not significantly different. At the same time, the thickness of the basal laminae was lower in the LF group than in the NF and LFT groups.

Table 2 shows the hepatic-function-related biochemical indicators. The TP, AST, ALT, AKP, ALB, and A/G values significantly increased in the LF group compared to in the NF and LFT groups (*p* < 0.05). There was no significant difference in hemolymph GLB among the three groups (*p* > 0.05). Correspondingly, the LF group significantly increased hepatopancreatic AST and ALT and decreased TP content more than the other groups (Figure 1B).

It was evident from the TUNEL staining diagram that more apoptosis occurred in the LF group than in the other two groups, as shown in Figure 1C. Statistically, the TUNEL-positive nuclei in the LF group were dramatically higher than in the NF and LFT groups (*p* < 0.05, Figure 1D). The LF group downregulated the protein level of the Bcl-2 and Bcl-2/Bax radio and upregulated the level of Bax compared to the other two groups (Figure 1E,F). In addition, the mRNA abundance of *Bax*, *Caspase-2*, *Caspase-3*, and *Caspase-8* presented a trend of increasing followed by decreasing among the three groups, and the Bcl-2 showed contrary trends (Figure 1G).

### 3.3. TTO Alleviates Metabolic Disorders Associated with LF Diet

The hemolymph metabolic indicators are shown in Table 2. The Glu and LDL-C content was significantly lower in the LF group than in the NF group (*p* < 0.05), and the Glu and LDL-C in the LFT group had no noticeable difference from the other groups. There was no significant difference in hemolymph TG, TC, and HDL-C among the three groups (*p* > 0.05).

**Figure 2:**
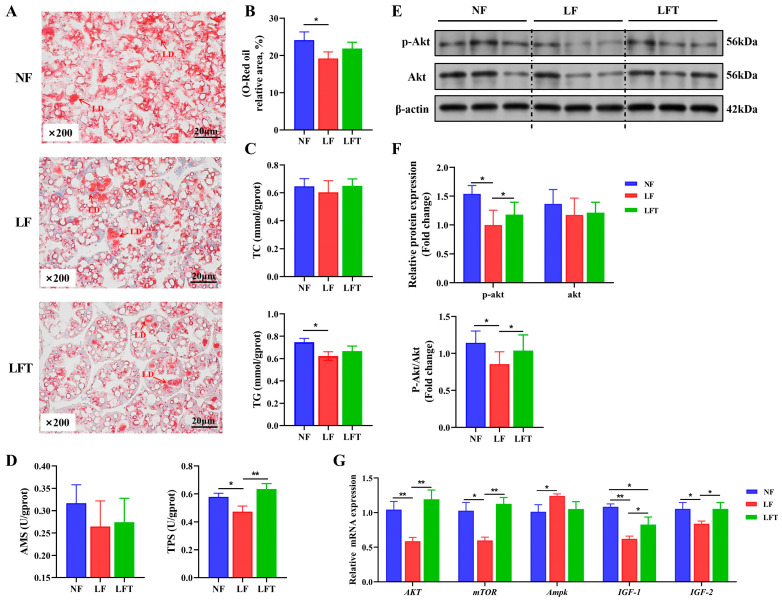
Effect of the three diets on the hepatopancreatic lipid energy and protein metabolism of *M. rosenbergii*. (**A**) The representative micrographs of Oil Red O staining (200× magnification) and relative area of lipid droplets; LD, Lipid droplet. (**B**) Relative area of lipid droplets. (**C**) TG and TC content in hepatopancreas. (**D**) AMS and TPS content in hepatopancreas. (**E**,**F**) The phosphorylation level of AKT protein was detected using Western blot analysis. (**G**) mRNA expression of energy metabolism-related genes. The asterisks indicate significant differences between the two groups. * *p* < 0.05, ** *p* < 0.01.

Figure 2A–C showed the status of lipid accumulation in the hepatopancreas. The hepatopancreatic lipid droplet area and TG content of the LF group were significantly lower than those of the NF group (*p* < 0.05). However, they had no statistical significance in the LFT group (*p* > 0.05). Meanwhile, the LF group had substantially lower TPS levels than the NF and LFT groups (*p* < 0.05), but the three groups had no significant difference in AMS activity (*p* > 0.05, Figure 2D).

In investigating the energy metabolism pathway AKT-mTOR, the protein level results showed that the LF group significantly inhibited the AKT-mTOR signaling pathway by downregulating the AKT phosphorylation level compared to the control. Furthermore, compared to the LF group, TTO could significantly activate the AKT-mTOR by enhancing the p-AKT/AKT ratio (Figure 2E,F). As a validation, the mRNA levels of the genes for the hepatopancreas are shown in Figure 2G; the LF group showed significantly lower *AKT*, *mTOR*, *IGF-1*, and *IGF-2* mRNA abundances than the other two groups (*p* < 0.05). The *IGF-1* mRNA level in the LFT group was significantly lower than in the NF group (*p* < 0.05). Additionally, the Ampk level in the LF group was significantly increased compared to that of the NF group (*p* < 0.05), but there was no significant difference in Ampk level between the LFT group and the NF or LF group (*p* > 0.05).

### 3.4. TTO Alleviates Innate Immunodeficiency from the LF Diet

Table 2 shows that the levels of the hemolymph T-SOD, GSH, and LZM presented a significant decrease in the LF group compared to the NF and LFT groups (*p* < 0.05), and the MDA showed the contrary. There was no significant difference in hemolymph CAT among the three groups.

**Figure 3:**
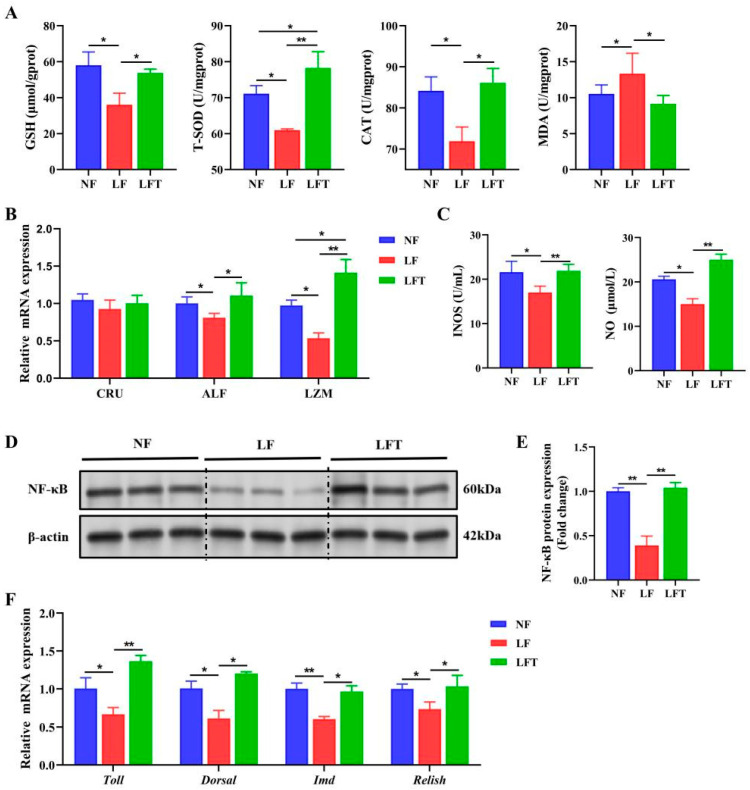
TTO alleviates innate immunodeficiency from the LF diet. (**A**) The regulation of antioxidant-related parameters. (**B**) mRNA expression of antibacterial peptide-related genes. (**C**) NO and INOS activity. (**D**,**E**) Western blot assays of NF-κB protein expression. (**F**) mRNA expression of prawns’ homologs of the components of NF-κB signal pathways-related genes. The asterisks indicate significant differences between the two groups. * *p* < 0.05, ** *p* < 0.01.

The hepatopancreatic antioxidant status is shown in Figure 3. The LF group had significantly lower levels of T-SOD, CAT, and GSH than the NF and LFT groups (*p* < 0.05). The LF group had significantly higher MDA levels than the other two groups (*p* < 0.05). In addition, the LFT group had significantly higher T-SOD levels than the NF group (*p* < 0.05). Figure 3B showed that the levels of two antimicrobial peptides, anti-lipopolysaccharide factor (ALF) and lysozyme (LZM), were significantly decreased in the LF group compared to the other two groups (*p* < 0.05). LZM levels were significantly higher in the LFT group than in the NF group (*p* < 0.05).

To examine the changes in the NF-κB pathway, we first measured the levels of INOS and NO in Figure 3C. The levels of INOS and NO were significantly downregulated in the LF group compared to the other two groups. The NF-κB signaling pathway was significantly inhibited in the LF group compared to the LFT group because the LF group significantly reduced the protein level of NF-κB compared to the LFT group (Figure 3D,E). The prawns’ homologs of the component’s mRNA levels are shown in Figure 3F. The LF group altered the *Toll*, *Dorsal*, *IMD*, and *Relish* mRNA expression. The expression levels of these four genes were significantly decreased compared to the other two groups (*p* < 0.05).

### 3.5. Alterations in the Physical Barrier and Microecological Structure of the Intestine by Three Diets

**Figure 4:**
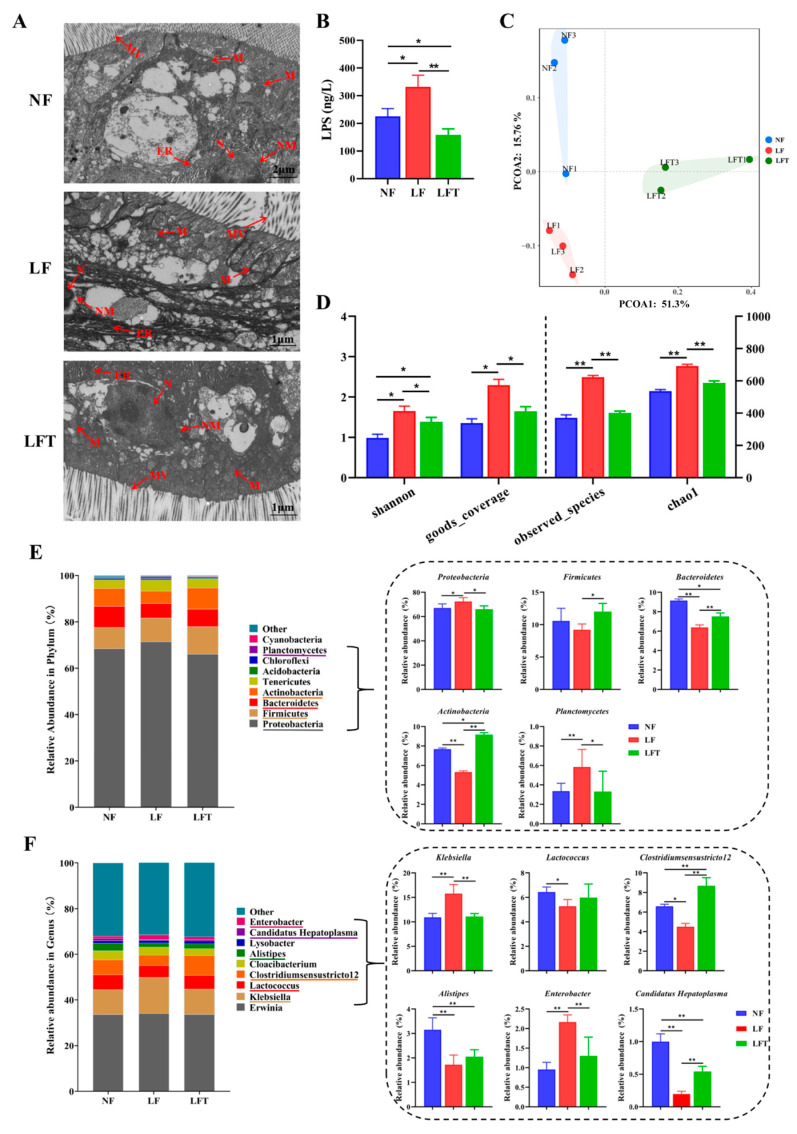
Effects of three diets on the structural diversity of intestinal microflora. (**A**) Microstructure of prawn intestine; MV, microvilli; ER, endoplasmic reticulum; N, nucleus; NM, nuclear membrane; M, mitochondria. (**B**) Hemolymph lipopolysaccharide (LPS) content. (**C**) Principal coordinate analysis of community. (**D**) Alpha diversity indices. (**E**,**F**) Microbiota composition at the phylum and genus level with relative abundance in the top ten. All data are expressed as the mean ± SEM. The asterisks indicate significant differences between the two groups. * *p* < 0.05, ** *p* < 0.01.

Figure 4A shows that TEM results showed intestinal microstructural damage in the LF group. The mitochondria in the LF group were found to be swollen, and the mitochondrial ridge was also broken and tended to dissolve; the endoplasmic reticulum matrix became irregularly arranged, microvilli ruptured, and the nuclear membrane was indistinct compared to the control group. After the supplementation of TTO in the LF diet, the endoplasmic reticulum recovered to its original state; the mitochondria, microvilli, and nuclear membrane almost recovered to the level of the NF control group.

In addition, the LF group had significantly elevated hemolymph LPS levels compared to the other two groups, and the LFT group had a significantly lower LPS level than the NF control (*p* < 0.05, Figure 4B). The Principal Coordinate Analysis (PCoA) results showed that the microbial composition in the NF, LF, and LFT groups was apparently different (Figure 4C). Meanwhile, to evaluate the diversity of the microbial community diversity, the Observed_species, Goods_coverage, Chao1, and Shannon indices were calculated based on the OTUs from each sample (Figure 4D). The Shannon, Goods_coverage, Observed_species, and Chao1 were significantly higher in the LF group than in the NF and LFT groups (*p* < 0.05). In addition, the Shannon results showed that the LFT group was significantly higher than the control group (*p* < 0.05).

The relative abundances of *Proteobacteria* and *Planctomycetes* in the LF group were significantly increased at the phylum level (*p* < 0.05) compared with the NF and LFT groups. At the same time, *Bacteroidetes* and *Actinobacteria* were significantly reduced (*p* < 0.05). The relative abundance of *Firmicutes* was significantly lower in the LF group than in the NF group (*p* < 0.05, Figure 4E). Additionally, the relative abundance of *Bacteroidetes* in the LFT groups was significantly decreased compared to the control group, and the level of *Actinobacteria* was the opposite (*p* < 0.05). At the genus level, as shown in Figure 4F, the relative abundance of *Klebsiella* and *Enterobacter* was extremely significantly increased in the LF group compared with the other two groups (*p* < 0.01), whereas the relative abundance of *Clostridiumsensustricto 12* and *Candidatus Hepatoplasma* was extremely significantly decreased (*p* < 0.01). The abundance of *Alistipes* and *Candidatus Hepatoplasma* was significantly lower in the LFT group compared to the control, and the *Clostridiumsensustricto 12* level was the opposite (*p* < 0.05). It is worth noting that the *Lactococcus* and *Alistipes* levels were significantly reduced in the LF group compared to the control group (*p* < 0.05).

### 3.6. Specific Microbial Compositional Differences

**Figure 5:**
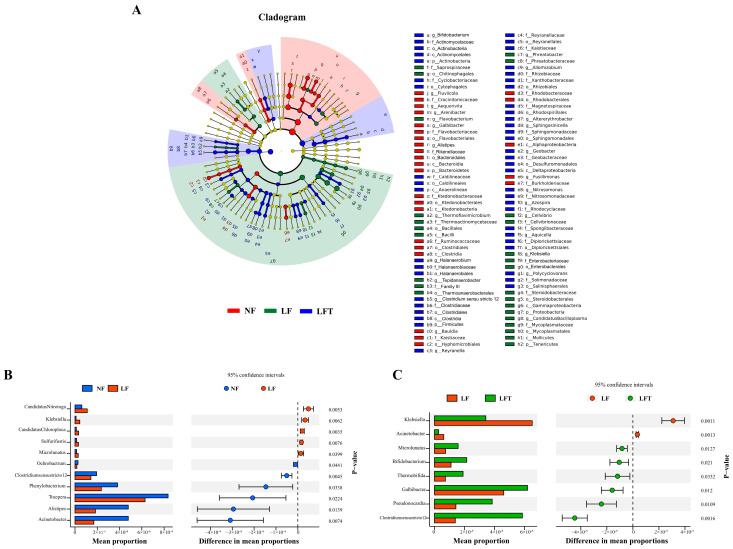
Analysis of differential intestinal microbes in three diets. (**A**) Microbial community biomarkers: LEfSe analysis identified the taxa with the most remarkable differences in abundance among the three taxa. Microbial comparation analysis on NF vs. LF (**B**) and LF vs. LFT (**C**) at genus level with *t*-test.

LEfSe was used to analyze the microbial communities under different diets, as shown in Figure 5. The most abundant phylotypes in the LF group, from phylum to genus level, were p_*Proteobacteria*, c_*Gammaproteobacteria*, o_*Enterobacterales*, f_*Enterobacteriaceae*, and g_*Klebsiella* compared to the NF and LFT groups (Figure 5A). The most abundant phylotypes in the LFT group included p_*Actinobacteria*, c_*Actinomycetales*, o_*Actinobacteria*, f_*Actinomycetaceae*, and g_*Bifidobacterium*, compared to the NF and LF groups.

Differences in microbial composition at the genus level were analyzed to understand further the effects of different diets on microbial composition in Figure 5B,C. STAMP analysis showed that the abundance of five genera (*Candidatus Nitrotoga*, *Klebsiella*, *Candidatus Chloroploca*, *Sulfurifustis*, *Microlunatus*) was increased, and that of six genera (*Ochrobactrum*, *Clostridiumsensustricto 12*, *Phenylobacterium*, *Truepera*, *Alistipes*, *Acinetobacter*) was significantly decreased in the LF group compared to the NF group (*p* < 0.05). In the LFT group, the abundance of six genera (*Clostridiumsensustricto 12*, *Pseudonocardia*, *Galbibacter*, *Thermobifida*, *Bifidobacterium*, *Microlunatus*) increased, and two genera (*Klebsiella*, *Acinetobacter*) decreased compared to the LF group (*p* < 0.05).

### 3.7. Differences in the Enrichment Function of Intestinal Microorganisms in Three Diets

**Figure 6:**
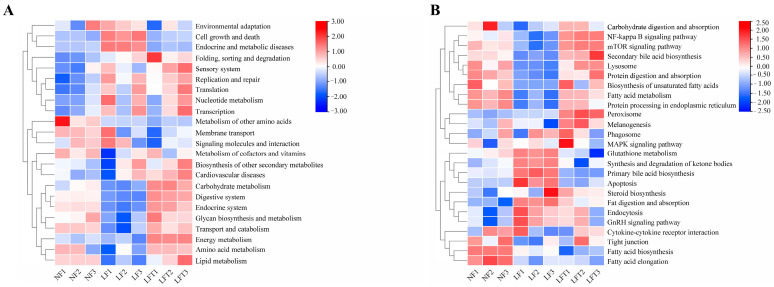
Effect of three diets on intestinal microbial functions. (**A**) Secondary functional annotation; (**B**) Tertiary functional annotation; red color represents upregulated function, and bule color represents downregulated function.

The prediction of the function of intestinal microorganisms in different diets by the Tax4Fun package (based on R software 4.3.0) is shown in Figure 6. The secondary function prediction (Figure 6A) showed that the LF group increased the abundance of cell growth and death and endocrine and metabolic diseases pathways compared to the other two groups, and decreased the abundance of carbohydrate metabolism, transport and catabolism, and amino acid metabolism compared to the LFT groups. However, the results of the LFT group showed a high abundance of energy metabolism compared to the NF and LF groups.

In addition, in tertiary function prediction (Figure 6B), the LF group predicted a lower abundance of the mTOR signaling pathway, secondary bile acid biosynthesis, lysosome, protein processing in the endoplasmic reticulum, and fatty acid metabolism, and a higher abundance of apoptosis and primary bile acid biosynthesis than the other two groups. The abundance of fatty acid biosynthesis and elongation was increased in the LF and LFT groups compared to the NF group. Simultaneously, the LF group had lower NF-κB signaling pathway levels than the LFT group.

### 3.8. Correlation Analysis of Hemolymph and Hepatopancreas Indicators with Intestinal Microbes

**Figure 7:**
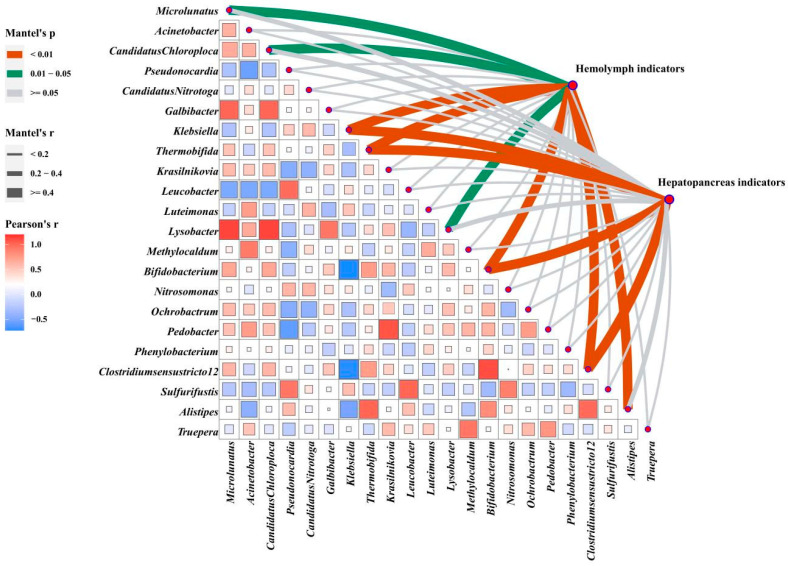
Correlation analysis of data sets. Multi-dimensional correlation analysis of screened differential microbes with hemolymph and hepatopancreas phenotype indicators. The microbes are compared in pairs with each other, with a color gradient and color block size denoting Spearman’s correlation coefficients (based on Euclidean distance). Hemolymph and hepatopancreas phenotypes are related to intestinal microbes using partial Mantel’s tests. Edge width corresponds to Mantel’s r statistic for the corresponding distance correlations and edge color denotes the statistical significance based on 9999 permutations.

The correlation analysis of intestinal microbes (based on Euclidean distances) and phenotypes (based on Manhattan distances) revealed that gut microflora played an essential role in shaping phenotypes (Figure 7). The abundances of *Klebsiella*, *Thermobifida*, *Clostridiumsensustricto 12*, and *Bifidobacterium* were highly relevant to both phenotypes (*p* < 0.01). Hemolymph indicators were significantly correlated with *Microlunatus*, *Candidatus Chloroploca*, and *Lysobacter* (*p* < 0.05) and significantly associated with *Alistipes* (*p* < 0.01).

A more specific presentation of Figure 7 and Figure S3A shows significant correlations between 17 measured hemolymph indicators, and 22 differential microbial genera were found through Pearson’s correlation analysis. The levels of AKB, ALB, MDA, AST, and LPS were significantly positively correlated with *Klebsiella* and almost negatively correlated with *Clostridiumsensustricto 12*, *Bifidobacterium*, and *Thermobifida*, while the hemolymph antioxidant enzymes (GSH, T-SOD, LZM) showed opposite trends (*p* < 0.05). In addition, the hemolymph’s GLU, TC, TG, and LDL-C content was significantly positively correlated with Alistipes (*p* < 0.05).

Figure S3B shows that hepatic enzyme activity (T-SOD, CAT, TP, AMS, AKP, NO, and INOS) and genes (*Bcl-2, AKT*, *mTOR*, *Toll*, *Dorsal*, *IMD*, *Relish*, *LZM*, and *ALF*) were almost positively correlated with *Thermobifida*, *Clostridiumsensustricto 12*, and *Bifidobacterium* and negatively correlated with *Klebsiella*. The apoptosis-related genes Bax, Caspase-2, Caspase-3, and Caspase-8 were positively correlated with *Klebsiella*, *Candidatus Nitrotoga*, and *Sulfurifustis*, but negatively correlated with *Microlunatus* and *Bifidobacterium* (*p* < 0.05). The *IGF-1* and *IGF-2* were positively correlated with *Alistipes* and *Methylocaldum* (*p* < 0.05). In addition, hepatopancreas injury-related indicators (MDA, ALT, AST) were significantly positively correlated with *Klebsiella* and negatively correlated with *Thermobifida*, *Clostridiumsensustricto 12*, and *Bifidobacterium* (*p* < 0.05).

## 4. Discussion

To develop low-cost and sustainable low-fish meal (LF) feed, soybean meal (SBM) can replace fish meal in the diet. However, an inappropriate replacement ratio results in a limitation of amino acids and ANFs, thus affecting the growth of aquatic animals [35]. Our results indicate that SBM replacing 40% fish meal (LF) reduced growth performance, leading to higher mortality and FCR in *M. rosenbergii*. Interestingly, the addition of TTO alleviated the growth inhibition without significant feed wastage and reduced it. In addition, although the addition of TTO resulted in a slight decrease in SR, WGR, and FCR compared to the control group, no significant difference was observed. These results suggest that the inclusion of the LFT diet in the production diet does not have negative effects on growth. Similar to our study, plant essential oils were found to have growth-promoting effects in aquaculture and did not have negative effects [36]. As nutrient uptake and pathogen resistance are generally achieved through enterohepatic circulation [37], we speculated that the inhibited growth and increased mortality of LF diets in our study were closely related to the physiological state of the hepatopancreas and intestine [38]. At the same time, it has also been reported that high levels of SBM diets caused pathological features in the enterohepatic tissues of *gibel carp* and *L. vannamei* [39,40]. Therefore, in this study, we used SBM to replace 40% fish meal (LF) as a side effect diet model. Subsequently, 200 mg/kg TTO was added to the LF diet (LFT) to investigate the protective effects of TTO against LF diet-induced enterohepatic injury in *M. rosenbergii*.

The hepatopancreas is the center of energy and lipid metabolism in crustaceans. The current results showed that the LF diet caused damage to the hepatopancreas, as indicated by a decrease in secretory cells and severe vacuolization in the microstructure in the LF group [14]. At the same time, the hepatic function indicators AST, ALT, and AKP were significantly elevated in the hepatopancreas and hemolymph in the LF group [41]. The significantly upregulated TP, ALB, and A/G levels in the LF group also reflected the unhealthy condition of the hepatopancreas, which was consistent with findings in *Eriocheir sinensis* [42,43]. However, the structural damage of the hepatopancreas and the level of liver injury indicators were alleviated by LFT, which could be attributed to the supplementation of TTO, inhibiting the activation of apoptosis caused by the LF diets [44]. The previous study showed that activated Akt/mTOR signaling could inhibit apoptosis. Specifically, phosphorylated AKT increased the Bcl-2/Bax ratio, which determines the direction of apoptosis (increasing the Bcl-2/Bax ratio inhibits apoptosis) [45]. In addition, the Akt/mTOR is also a pivotal pathway that promotes histiocyte growth and differentiation and even regulates energy (glucose and lipid) metabolism [46]. In this study, LFT alleviated AKT/mTOR expression and AKT phosphorylation inhibited by LF, suggesting that LFT could alleviate apoptosis and energy metabolism disorder caused by LF.

In energy metabolism, trypsin plays an important role in protein digestion, releasing amino acids and glucose to provide energy for the organism [47,48]. In the present study, decreased trypsin and blood glucose levels in the hepatopancreas revealed an LF-induced energy metabolism disorder. Consistently, hepatic weight, HSI, hepatic lipids, and blood lipids were all decreased in the LF group, indicating that hepatopancreas dysplasia caused hepatic atrophy and lipid loss [49]. Meanwhile, the polypeptide hormones IGF-1 and IGF-2, as well as the downstream Akt/mTOR signaling pathway, were downregulated in the LF group, indicating that LF inhibited hepatic development and growth [50]. After TTO was added, IGF-1 and IGF-2 levels were upregulated, restoring energy metabolism and hepatopancreas development to the control levels. In conclusion, TTO supplementation reversed the hepatopancreas dysplasia and damage caused by SBM replacing the fish-meal diet.

Crustaceans rely on innate immunity (non-specific immunity) to resist damage from pathogens and other noxious agents. As a critical immune organ in crustaceans, the hepatopancreas exhibits non-specific immune deficiencies when hepatopancreatic damage and energy metabolism disorders occur [51]. Antioxidant capacity is a part of innate immunity, which depends on the ability of antioxidants to scavenge free radicals or other harmful substances, such as GSH, SOD, CAT, and GPX. Consistent with other studies, decreased SOD, GSH, CAT, and increased MDA levels in the hepatopancreas and hemolymph in this study suggest that LF reduces antioxidant capacity [7]. However, TTO alleviated the antioxidant dysfunction through non-specific immune signaling pathways, such as the NF-κB/NO pathway and the homologous proteins Dorsal and Relish, and the ligand proteins Toll and IMD for Dorsal and Relish [27,52]. In addition, antibacterial peptides (AMPs) from crustin (Cru), lysozyme (LZM), and anti-lipopolysaccharide factor (ALF) are important for antioxidant resistance and innate immunity when induced by Dorsal and Relish [53,54]. In the present study, TTO activated the expression of ALF, LZM, and hemolymph LZM. Therefore, the addition of TTO improved the non-specific immune ability and antioxidant capacity through the NF-κB/NO and NF-κB/AMPs pathways.

In addition to digestion and absorption, the shrimp intestine also functions as a critical immune organ. There are trillions of gut bacteria in the intestine, but the disruption of intestinal flora leads to an increased abundance of pathogenic bacteria, ultimately resulting in intestinal damage. In addition, the intestinal flora participates in several biological reactions in the host, of which intestine–hepatic connections are the most meaningful [55]. In the LF group, we found markedly affected intestinal microbial composition and diversity, including a significant increase in the abundance of *Proteobacteria*. All bacteria in this phylum are Gram-negative and are the primary source of plasma LPS. In our study, hemolymph LPS replacement showed that LPS entered the hepatopancreas via the enterohepatic circulation and promoted the initiation and progression of hepatopancreatic injury [56]. It is worth noting that the abundance of *Firmicutes* and *Actinobacteria* was significantly increased by the addition of TTO. The *Firmicutes* included the vast majority of lactic acid bacteria and Clostridium genera, such as *Lactobacillus*, *Clostridiumbutyricum*, and *Leuconostoc*, which are generally considered to be probiotics for intestinal health [57]. The phylum *Actinobacteria* produced short-chain fatty acids (SCFAs) associated with oligosaccharide-fermenting bacteria such as *Bifidobacterium* [58]. Thus, the recovery of *Firmicutes* and *Actinobacteria* abundance following TTO intervention suggests that TTO has the potential to positively improve the intestinal flora.

Tax4Fun microbial function analysis is an effective method to identify microbial functions and has been used in many studies [59]. Through Tax4Fun analysis, we found dysregulated gut microbial functions in the energy metabolism, cell apoptosis, and NF-κB pathway. However, the mechanisms between these gut microbiota and the critical pathway are still unclear. Therefore, we performed Pearson correlation analyses to uncover the relationship between the three different microbial genera, the hemolymph, and the hepatopancreatic indicators.

The results showed that *Clostridium sensu stricto 12* and *Thermobifida* were positively correlated with antioxidant indicators and negatively correlated with hepatic damage. *Clostridium sensu stricto* and *Thermobifida* are both beneficial microorganisms, but there is no detailed research indicating their direct relationship with hepatic injury and antioxidant activity. We speculated that they might be mediated through two pathways; on the one hand, some metabolites produced by *Clostridium sensu stricto* and *Thermobifida* regulate the immune system by stimulating the secretion of mucin proteins in intestinal mucosal epithelial cells to improve intestinal mucosal barrier function. Another possible mechanism is that their metabolites may contain a variety of antioxidants that could scavenge free radicals and reduce hepatic oxidative damage, such as GSH and SOD. Based on the changes in non-specific immune competence in *M. rosenbergii*, we believe that the first speculation is the most likely. Further studies are needed.

Research has shown that *Klebsiella* is often regarded as a pernicious bacterium, leading to intestinal barrier damage, apoptosis, and defects in the immune signaling pathway [60]. In our study, *Klebsiella* showed a negative correlation with antioxidant and immune-related indicators, while being positively correlated with hypohepatia and apoptosis indicators, suggesting that increased *Klebsiella* abundance by LF leads to immune suppression and oxidative damage. The pathogenic factor LPS expressed by *Klebsiella* also caused the onset and exacerbation of hepatic disease, leading to hypohepatia [61]. A significant negative correlation has also been found between the AMPs gene and *Klebsiella*, indicating that a decrease in AMPs in the LF group may lead to *Klebsiella* colonization in the intestine [62]. *Bifidobacterium* is a probiotic in the gut that is critical to improve hepatic development and function [63,64]. It is worth noting that *Bifidobacterium* has completely opposite effects to *Klebsiella*, suggesting that *Bifidobacterium* positively enhances growth performance and immune function. In addition, as an upstream regulator of NF-κB, the activation of Akt/mTOR by *Bifidobacterium* upregulates the expression of NF-κB homologous genes (Relish/Imd, Toll/Dorsal), which may ultimately achieve antioxidant activity through NF-κB/NO. At the same time, AMPs genes activated by NF-κB transcription factors can induce immune responses [65], further demonstrating that *Bifidobacterium* could enhance non-specific immunity [66]. Additionally, *Alistipes* could improve the metabolic state in the gastrointestinal tract, increasing the host’s ability to use glucose for energy while promoting increased fatty acid synthesis [67]. Interestingly, glucose and lipid levels, IGF-1, and IGF-2 were positively correlated with *Alistipes* in this study. Therefore, *Alistipes* may promote increased glucose utilization, lipid synthesis, and IGF-1 and IGF-2 levels to promote growth. Our results showed that the growth and immunosuppression produced by replacing fishmeal with SBM were correlated with the increase in *Klebsiella* in the intestine. At the same time, an improvement after TTO supplementation can be achieved by probiotics such as *Clostridium sensu stricto 12*, *Thermobifida*, *Bifidobacterium*, etc., to improve intestinal function.

## 5. Conclusions

**Figure 8:**
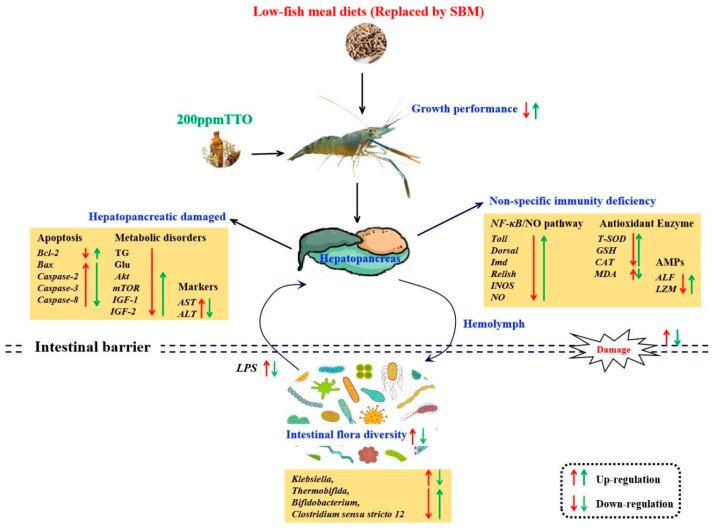
Mechanisms of regulation of the hepatopancreas–intestine axis and intestinal microbiota after LF diet and 200 mg/kg TTO supplementation (LFT). The red arrows indicate the regulatory effects of the LF diet; the green arrows indicate the regulatory effects of the LFT diet. Arrows pointing upwards indicate significant upregulation or improvement and arrows pointing downwards indicate significant downregulation or inhibition.

In conclusion, the LF diet resulted in significant growth inhibition. Although a small negative effect on growth performance was observed in the LFT diet, it was not very severe considering its non-significance and several positive health benefits. The growth inhibition and hepatopancreas damage caused by the LF diet may be related to metabolic disturbances and non-specific immunodeficiency, which were caused by increased levels of LPS by *Klebsiella*. The positive regulation of non-specific immunity and energy metabolism in the hepatopancreas of *M. rosenbergii* by the addition of 200 mg/kg TTO may depend on the combined effects of metabolites from different microorganisms (*Clostridium sensu stricto 12*, *Thermobifida*, *Bifidobacterium*) on the AKT/mTOR and NF-κB/NO pathways (Figure 8).

## Figures and Tables

**Table 1:** Effects of dietary TTO levels on the growth evaluation of *M. rosenbergii*.

Index	Groups			*p*-Value
	NF	LF	LFT	
Survival rate (SR, %)	88.75 ± 3.70 ^b^	82.50 ± 2.20 ^a^	85.00 ± 1.44 ^ab^	0.033
Weight gain rate (WGR, %)	1389.91 ± 71.88 ^b^	1246.84 ± 33.72 ^a^	1326.25 ± 57.86 ^b^	0.023
Specific growth rate (SGR, %/day)	6.58 ± 0.05 ^b^	6.22 ± 0.12 ^a^	6.49 ± 0.26 ^b^	0.019
Feed conversion ratio (FCR)	1.24 ± 0.08 ^a^	1.44 ± 0.02 ^b^	1.20 ± 0.02 ^a^	0.036
Hepatosomatic index (HSI, %)	10.63 ± 1.21 ^b^	6.86 ± 0.78 ^a^	9.74 ± 0.56 ^b^	0.009

Note: data are expressed as means with SEM (*n* = 3). Values with different superscripts are significantly different according to one-way ANOVA (*p* < 0.05).

**Table 2:** Glucolipid metabolism and biochemical indicators in the hemolymph of *M. rosenbergii*.

Hemolymph Parameters	Groups			*p*-Value
	NF	LF	LFT	
Glucolipid metabolism				
Glu (mmol/L)	9.72 ± 1.16 ^b^	7.86 ± 0.76 ^a^	8.65 ± 0.92 ^ab^	0.032
TG (mmol/L)	0.68 ± 0.29	0.61 ± 0.29	0.65 ± 0.15	0.105
TC (mmol/L)	0.75 ± 0.33	0.70 ± 0.13	0.71 ± 0.14	0.154
LDL-C (mmol/L)	0.28 ± 0.05 ^b^	0.15 ± 0.04 ^a^	0.21 ± 0.04 ^ab^	0.036
HDL-C (mmol/L)	0.40 ± 0.04	0.46 ± 0.03	0.42 ± 0.05	0.326
Biochemical				
TP (g/dL)	17.55 ± 1.14 ^a^	21.64 ± 2.09 ^b^	18.43 ± 1.41 ^a^	0.007
ALB (g/dL)	7.82 ± 0.28 ^a^	9.70 ± 0.24 ^b^	8.07 ± 0.09 ^a^	0.017
GLB (g/dL)	15.58 ± 0.17	14.97 ± 0.06	15.06 ± 0.15	0.268
ALB:GLB (A/G, %)	50.23 ± 2.11 ^a^	64.79 ± 1.81 ^b^	53.63 ± 0.48 ^a^	0.019
AST (U/L)	38.14 ± 4.27 ^a^	64.73 ± 6.72 ^b^	43.61 ± 3.74 ^a^	0.009
ALT (U/L)	12.32 ± 2.10 ^a^	18.62 ± 2.81 ^b^	13.45 ± 1.65 ^a^	0.016
AKP (U/L)	2.84 ± 0.17 ^a^	5.05 ± 0.24 ^b^	3.44 ± 0.23 ^a^	0.026
Enzyme activity				
T-SOD (U/mL)	57.66 ± 2.88 ^b^	50.38 ± 3.14 ^a^	57.42 ± 2.65 ^b^	0.002
CAT (U/mL)	22.59 ± 1.21	20.81 ± 3.08	23.16 ± 1.49	0.158
GSH (nmol/mL)	87.23 ± 4.21 ^b^	51.71 ± 5.73 ^a^	92.65 ± 6.59 ^b^	0.001
MDA (nmol/mL)	15.74 ± 1.44 ^a^	20.8 ± 1.62 ^b^	16.08 ± 2.13 ^a^	0.019
LZM (U/mL)	175.65 ± 21.40 ^b^	125.33 ± 13.93 ^a^	185.65 ± 31.24 ^b^	0.001

Note: data are expressed as means with SEM. Values with different superscripts are significantly different according to one-way ANOVA (*p* < 0.05).

## Data Availability

Data are contained within the article.

## Supplementary Materials

The following supporting information can be downloaded at: https://www.mdpi.com/article/10.3390/antiox12101879/s1, Table S1. The formulation and proximate composition of the experimental diet. Table S2. Basic growth indicators. Table S3. qPCR primer. Figure S1. The optimum level of TTO supplementation based on second-order polynomial analysis on weight gain rate (WGR) and specific growth rate (SGR) of *M. rosenbergii*. Figure S2. The optimum level of TTO supplementation based on second-order polynomial analysis on antioxidant indicators (T-SOD, MDA) of *M. rosenbergii*. Figure S3. Heat map of correlation of hemolymph and hepatopancreas measures with intestinal microbial genera.
