# Supplementary material for: Tea Tree Oil Improves Energy Metabolism, Non-Specific Immunity, and Microbiota Diversity via the Intestine–Hepatopancreas Axis in Macrobrachium rosenbergii under Low Fish Meal Diet Administration
Source: Antioxidants (Basel). 2023 Oct 19;12(10):1879. doi: 10.3390/antiox12101879 (PMC10604904; doi:10.3390/antiox12101879)
Supplement: Supplementary file 1 [file antioxidants-12-01879-s001.zip › antioxidants-2642307-supplementary.pdf]

Table S1. The formulation and proximate composition of the experimental diet

Ingredients (g)	NF	LF	LFT
Fish meal[1]	250.00	150.00	150.00
Soybean meal[1]	190.00	290.00	290.00
Chellocken meal[1]	30.00	30.00	30.00
Blood globulin powder[1]	20.00	20.00	20.00
Squid extract[1]	30. 00	30.00	30.00
Shrimp meal[1]	40.00	40.00	40.00
Repeseed meal[2]	155.00	155.00	155.00
α-starch[1]	165.00	165.00	165.00
Soybean oil[2]	10.00	10.00	10.00
Soy lecithin oil[1]	10.00	10.00	10.00
Cholesterol[4]	3.00	3.00	3.00
Ecdysone[1]	0.10	0.10	0.10
MCP	20.00	20.00	20.00
Premix[3]	10.00	10.00	10.00
Choline chloride[4]	10.00	10.00	10.00
Bentonite[4]	6.90	6.90	4.90
Carboxymethocel	50.00	50.00	50.00
Tea Tree Oil(10%)	0.00	0.00	2.00
Total	1000.00	1000.00	1000.00
Proximate analysis (%)			
Moisture	10.05	10.74	10.33
Crude protein	38.28	37.83	37.91
Crude lipid	9.64	9.21	9.16
Ash	10.67	10.81	10.47

[1] Obtained from Jiangsu Fuyuda Food Products Co., Ltd., Yangzhou, China;

[2]Obtained from Hulunbeier Sanyuan Milk Co., Ltd., Inner Mongolia,China;

[3]Premix supplied the following minerals (g kg^{-1}) and vitamins (IU or mg kg^{-1}): $CuSO_4{\cdot}5H_2O$, 2.0 g; $FeSO_4{\cdot}7H_2O$, 25 g; $ZnSO_4{\cdot}7H_2O$, 22 g; $MnSO_4{\cdot}4H_2O$, 7 g; Na_2SeO_3, 0.04 g; KI, 0.026 g; $CoCl_2{\cdot}6H_2O$, 0.1 g; Vitamin A, 900,000 IU; Vitamin D, 200,000 IU; Vitamin E, 4500 mg; Vitamin K_3, 220 mg; Vitamin B_1, 320 mg; Vitamin B_2, 1090 mg; Vitamin B_5, 2000 mg; Vitamin B_6, 500 mg; Vitamin B_{12}, 1.6 mg; Vitamin C, 5000 mg; Pantothenate, 1000 mg; Folic acid, 165 mg. Vitamin and mineral additives were provided by Wuxi Hanove Animal Health Products Co., Ltd.

[4] Obtained from Freshwater Fisheries Research Center, Chinese Academy of Fishery Sciences.

Table S2. Basic growth indicators

Index	Groups		
	NF	LF	LFT
Initial weight (g)	0.28±0.01	0.28±0.01	0.29±0.01
Final weight (g)	3.91±0.17	3.43±0.43	3.90±0.46
Hepatopancreas weight (g)	0.41±0.04	0.24±0.02	0.38±0.03
Dry feed intake (g / per shrimp)	9.37±0.08	9.13±0.17	9.21±0.14

Table S3. qPCR primer

Gene	Primer	Sequences (5′-3′)	Accession number
β-actin	F	TCCGTAAGGACCTGTATGCC	AY651918.2
	R	TCGGGAGGTGCGATGATTTT	
BCL-2	F	GTATAAAGTGCGTTCGCCCC	OR602687
	R	AATGACGTGGTCGTGGAAGT	
Bax	F	GTTCGCCCCTCTGCTCAATA	OR602688
	R	CAGTCTTTCATGTGCGGCG	
Caspase-2	F	ACCTGGTTACTATTCACGC	OR602689
	R	TAAGATCATATTTTTGGGC	
Caspase-3	F	AGGGGATAAAACCGATGGAG	KF878972.1
	R	CCCACATGACGAGCATATCA	
Caspase-8	F	CACGGCAAAACTGGAGGAG	OR602691
	R	CGGCGAGAATCAAAAGACA	
Akt	F	GGTCCAAACATGCTCAACCAA	OR602692
	R	AGGTGTTCCCTACTTTCACTTGC	
mTOR	F	GAGACTTCTGCCACGACGAA	OR602693
	R	CAAAACGGGCACCAACCAAT	
Ampk	F	TGAAAGGGCGCTAAGTGGAG	OR602694
	R	ACTCGCACATGGAAAGGGTT	
IGF-1	F	AATAAAAACGGTGACAGAG	OR602695
	R	TGGAAATTCAAATAATGAG	
IGF-2	F	CCCCCTTTGTTTGTGTTTT	OR602696
	R	AGCTCGGGTAGATGTGCAT	
Toll	F	TCAGTAGCGACACCATGCAG	KX610955.1
	R	CGAGCCTTCGAGGAACACTT	
Drosal	F	TCAGTAGCGACACCATGCAG	KX219631.1
	R	CGAGCCTTCGAGGAACACTT	
IMD	F	CGACCACATTCTCCTCCTCCC	OR602697
	R	TTCAGTGCATCCACGTCCCTC	
Relish	F	GATGAGCCTTCAGTGCCAGA	KR827675.1
	R	CCAGGTGACGCCATGTATCA	
CRU	F	GCAGGTGACGGTTGAGGA	OR602698
	R	ATGCGACTGACTGGTGGA	
ALF	F	GGAAGGCAGACATTGGACC	OR602699
	R	GCAGACGCAGAAGGAAGG	
LZM	F	CCGTCATCTTCGCCTTGGTT	OR602700
	R	TGCCTGTTTGGGTCATCGTTC	

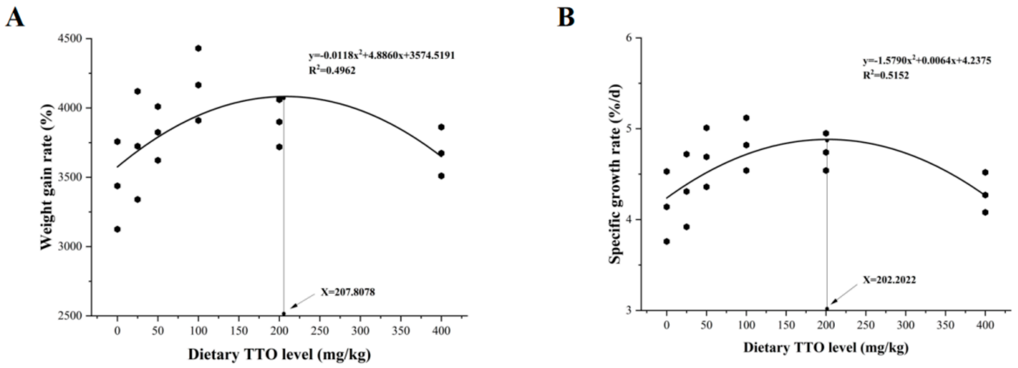

Figure S1. The optimum level of TTO supplementation based on second order polynomial analysis on (A) weight gain rate (WGR) and (B) specific growth rate (SGR) of *M. rosenbergii.* Note: Data are mean values of 3 replicates expressed as mean ± SEM.

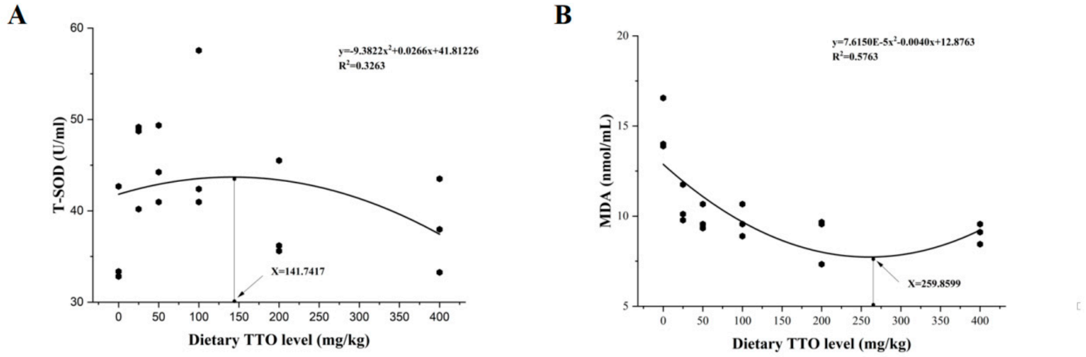

Figure S2. The optimum level of TTO supplementation based on second order polynomial analysis on antioxidant indicators of *M. rosenbergii.* (A) Total superoxide dismutase (SOD); (B) Malondialdehyde (MDA). Note: Data are mean values of 3 replicates expressed as mean ± SEM.

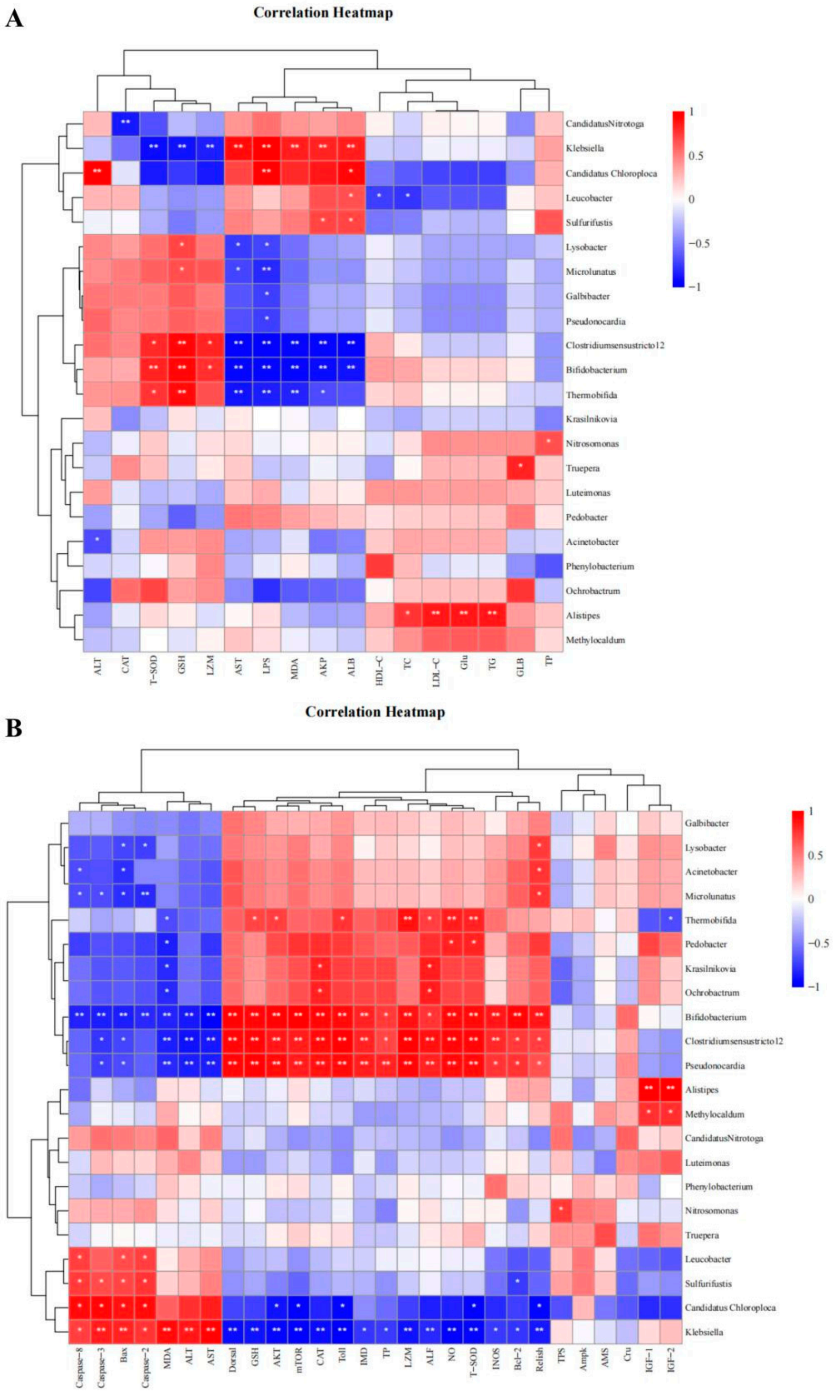

Figure S3. Correlation of hemolymph and hepatopancreas measures with intestinal microbial genera. (A) Heatmaps of hemolymph parameters and microbiota genera abundance; (B) Heatmaps of hepatopancreas indicators and microbiota genera abundance. Red represents a positive correlation; blue represents a negative–positive correlation. *$p < 0.05$, **$p < 0.01$.
